# Comparison of Global Metabolite for Growing Pigs Fed at Metabolizable Energy Requirement for Maintenance

**DOI:** 10.3389/fvets.2022.917033

**Published:** 2022-07-08

**Authors:** Hu Liu, Yifan Chen, Wenhui Wang, Zhaoning Jiang, Xi Ma, Fenglai Wang

**Affiliations:** ^1^State Key Laboratory of Animal Nutrition, College of Animal Science and Technology, China Agricultural University, Beijing, China; ^2^College of Animal Science and Technology, Hebei Agricultural University, Baoding, China

**Keywords:** energy metabolism, growing pigs, maintenance, metabolomics, venous catheter

## Abstract

Though the energy requirement for maintenance is an important part of net energy system, little is known of the metabolic characteristics of maintenance energy expenditure. This study was investigated the effect of feeding level at metabolizable energy requirement for maintenance (FLM) on plasma metabolites in growing pigs. Ten barrows (22.5 ± 0.5 kg BW) were kept in metabolism crates and catheterized in the precaval vein during adaptation period. Pigs were fed a corn-soybean meal diet at 782 kJ ME/kg BW^0.6^·d^−1^ during d 1 to 8 and then were refeeding at 2,400 kJ ME/kg BW^0.6^·d^−1^ on d 9. Plasma samples of each pig were collected by catheter on the morning of d 1, 3, 5, 7, 9, and 10, respectively, for metabolomics testing. Results showed that the concentration of plasma urea nitrogen decreased under FLM (*p* < 0.01) and increased significantly after refeeding (*p* < 0.01). The concentration of total cholesterol, high-density lipoprotein, low-density lipoprotein, and albumin in plasma were decreased significantly after refeeding (*p* < 0.01). Eleven identified compounds were up-regulated and six ones were down-regulated under FLM. In conclusion, the energy metabolism of growing pigs was relatively stable after 4 days of feeding at FLM.

## Introduction

The energy metabolism of maintenance in pigs means that when the external environmental conditions are suitable, the energy supplied by the feed meets the basal requirements for supporting body function, body temperature and necessary activity, and the metabolism of energy-carrying substances in the body's tissues is in a state of dynamic equilibrium (1). In this status, the energy requirements of growing pigs are energy requirements for maintenance (2). It has been reported in the literature that the energy requirement for maintenance in growing pigs was in the range of 720–1,122 kJ ME/kg BW^0.6^·d^−1^ (3– 5).

In the previous studies of our laboratory, the energy requirement for maintenance in growing pigs was estimated at 782 kJ ME/kg BW^0.6^·d^−1^ through the body weight regression method (6). Obviously, it can be known that the energy requirement for maintenance in growing pigs was accounted for 30 to 35% of the total energy requirement, which indicated that the state of maintenance in growing pigs was actually a manifestation of feed restriction.

Feed restriction was often applied to a certain stage of the early growth or rapid growth period of animals. By controlling the nutrient and energy intake of animals, the deposition of protein and fat in body can be restrained or even suspended so that the animal is maintained or slow growth (7). There were many methods of feed restriction used in previous studies, mainly including artificial control of feed intake, diet dilution method and nutrient restriction method (8, 9). Among them, artificial control of feed intake was relatively common. It was a means to limit the metabolizable energy feeding level of growing pigs by cutting down the amount of the balanced diet (10). Studies have shown that under the condition of feed restriction, growing pigs reduced the total energy expenditure and basal metabolic rate by 30% through the body's nerve and endocrine regulation (11). Meanwhile, Krueger et al. also reported that the energy from body's carbohydrate oxidation decreased and the energy from fat oxidation increased. However, little is known about the regulate mechanism of energy metabolism in growing pigs under the state of maintenance.

Metabolomics can explore the dynamic response of all metabolites caused by environmental stimuli, pathological disturbances or genetic mutation in the life system (12). Compared with other omics techniques, metabolites are at the terminal of the regulation of gene and protein expression activities in biological systems, which contains direct and comprehensive metabolic marker information that reflects the body's physiological phenotype. Therefore, metabolomics was often used to explore the time effects of experimental treatments on the body tissue or plasma-related metabolic spectrum (13). Jiang et al. (14) used metabolomics technology to study the effect of different time of high-fat diet intake on hamster plasma metabolites. Their results showed that plasma metabolites at different time points can reflect the occurrence process of hyperlipidemia in hamsters from normal physiological to pathological conditions.

Therefore, the current experiment used artificially controlled feed intake to meet the energy requirement for maintenance in growing pigs. In that situation, the changes in plasma metabolites of growing pigs at feed restriction and refeeding were detected and the time required for growing pigs to the status of maintenance were verified.

## Materials and Methods

### Animal, Diets and Experimental Procedures

The animal procedures described in this experiment were approved by the Institutional Animal Care and Use Committee of China Agricultural University (No. AW11102202-1-2). A total of ten growing barrows (Duroc × Landrace × Yorkshire) with similar BW (22.5 ± 0.5 kg) were selected from the Fengning Swine Research Unit of China Agricultural University (Hebei, China). The basal diet (Table 1) was formulated based on corn and soybean meal to meet or exceed nutrient requirements according to the NRC 2012 (15).

**Table 1:** Composition and nutrient analysis of the experimental diet (%, as-fed basis).

**Items**	**Basal diet**
**Ingredients**	
Corn	75.34
Soybean meal	21.00
Dicalcium phosphate	1.00
Limestone	0.80
Salt	0.35
L-Lys HCl	0.50
DL-Met	0.11
L-Trp	0.10
L-Thr	0.30
Vitamins and minerals premix ^a^	0.50
**Nutrient levels** ^**b**^	
DM	88.79
GE, MJ/kg	16.45
ME, MJ/kg	14.02
CP	16.41
EE	3.82
NDF	9.77
ADF	3.69
Calcium	0.68
Total phosphorus	0.56
**SID amino acids**	
Lys	0.99
Met	0.29
Trp	0.22
Thr	0.63

*^a^Premix supplied the following nutrients per kilogram of diet: vitamin A, 5,512 IU; vitamin D3, 2,200 IU; vitamin E, 30 IU; vitamin K3, 2.2 mg; vitamin B12, 27.6 μg; thiamine, 1.5 mg; riboflavin, 4.0 mg; pantothenic acid, 14.0 mg; niacin, 30.0 mg; choline chloride, 400.0 mg; folic acid, 0.7 mg; pyridoxine, 3.0 mg; biotin, 44.0 μg; Mn, 40.0 mg; Fe, 75.0 mg; Zn, 75.0 mg; Cu, 100.0 mg; I, 0.3 mg; Se, 0.3 mg. ^b^ Values of ME and SID amino acids were calculated according to the NRC (2012). Other nutrient levels were analyzed value*.

Pigs were individually housed in stainless-steel metabolism cages and adapted to the cages and diet for 7 days. All pigs were anesthetized and an indwelling catheter was inserted into the anterior vein before the experiment. After 3-day recovery, pigs were weighed at the beginning of the experiment and fed 780 kJ ME/kg BW^0.6^ d^−1^ during d 1 to d 8, which approximated to energy requirement for maintenance (FLM). Then these pigs were refeeding at 2,400 kJ ME/kg BW^0.6^ d^−1^ at d 9, which approximated to the *ad libitum* feed intake. Blood samples of each pig were collected into heparin anticoagulation tubes before feeding during the experimental period, and then the extension tube was filled with heparinized saline to permit sampling from outside the pens. Pigs were fed the basal diet twice daily in two equal meals at 08.00 and 16.00 hr. during the adaptation and experimental periods. Each crate was fitted with a one-hole feeder and a low-pressure nipple drinker and located in an environmentally controlled room with the temperature maintained at 22 ± 2°C.

### Sample Preparation, Chemical and UPLC-MS Analysis

Chemical analyses of all samples were conducted in duplicate. The diet was analyzed including dry matter (DM), crude protein (CP), calcium, total phosphorus (16) and ether extract (EE) (17). Neutral detergent fiber (NDF) and acid detergent fiber (ADF) were measured with filter bags using a Fiber Analyzer (Ankom Technology, Macedon, NY, USA). Gross energy (GE) was determined by an automatic adiabatic oxygen bomb calorimeter (Parr 1281, Automatic Energy Analyzer; Moline, IL, USA) according to previous study (18).

Blood samples were centrifuged (Biofuge22R; Heraeus, Hanau, Germany) at 3,000 × g for 10 min at 4°C, then the supernatant was transferred to storage tubes and stored at−80°C. After the frozen samples were thawed at 4°C, plasma levels of triglyceride, total cholesterol, high-density lipoprotein, low-density lipoprotein, total protein, albumin, and urea nitrogen were quantified using a biological analyzer (7600 Automatic Biological Analyzer; Hitachi, Tokyo, Japan) at the Kangjia Hongyuan Biotech Company (Beijing, China). Sample preparation for metabolomics, UPLC-MS analysis and data mining and processing according to previous study (1).

### Statistical Analysis

Data generated in the present experiment were analyzed using the GLM procedure of SAS (SAS Inst. Inc., Cary, NC). The individual pig was used as the experimental unit for all response variables in the model, which included feeding duration as the main effect. The LSMEANS statement were used to separate the treatment means and multiple comparison. Results were considered significant at *p* < 0.05 and considered as trends at 0.05 < *p* < 0.10. Cluster analysis and boxplot analysis of identified differential compounds were achieved using the R software package (R Development Core Team (2017), version 3.4.1). Pathway analysis of metabolite profiles was carried out using MetaboAnalyst 3.0 (http://www.metaboanalyst.ca).

## Results

### Effect of FLM on Plasma Metabolites in Growing Pigs

The effect of FLM on plasma biochemical parameters in growing pigs were shown in Table 2. Under the condition of FLM, the plasma urea nitrogen of growing pigs gradually decreased with the extension of feeding time, while the concentration of urea nitrogen increased significantly after refeeding (*p* < 0.01). Total cholesterol, high-density lipoprotein, low-density lipoprotein, and albumin in plasma were not affected by FLM, but the concentration of these index decreased significantly after refeeding (*p* < 0.01). Plasma triglycerides and total protein of growing pigs were not affected during the experiment. There was no significant difference in the plasma biochemical indexes of growing pigs on the 4th to 8th day during FLM.

**Table 2:** Effects of feeding level at metabolizable energy for maintenance on the concentration of plasma biochemical parameters in growing pigs.

**Items**	**Feeding time, d** ^**1**^						**SEM**	***P* value**
	**0**	**2**	**4**	**6**	**8**	**9**		
Replicates	10	10	10	10	10	10		
Triglyceride, mmol/L	0.25	0.40	0.32	0.30	0.26	0.31	0.04	0.10
Total cholesterol, mmol/L	2.16^ab^	2.35^a^	2.40^a^	1.96^ab^	2.13^ab^	1.71^b^	0.12	<0.01
High-density lipoprotein, mmol/L	0.45^a^	0.42^ab^	0.37^abc^	0.29^bc^	0.32^abc^	0.25^c^	0.03	<0.01
Low-density lipoprotein, mmol/L	1.17^ab^	1.31^ab^	1.51^a^	1.21^ab^	1.33^ab^	1.06^b^	0.08	0.01
Total protein, g/L	69.40	70.30	75.39	74.06	75.86	73.68	2.00	0.17
Albumin, g/L	36.44^a^	37.11^a^	37.47^a^	35.99^a^	36.18^a^	31.40^b^	0.76	<0.01
Urea nitrogen, mmol/L	2.39^ab^	3.56^a^	1.86^bc^	1.83^bc^	1.94^bc^	3.65^a^	0.34	<0.01

*^1, a, b, c^represents significant difference, p < 0.05*.

### Plasma Metabolic Profiling Based on UPLC-HRMS

**Figure 1:**
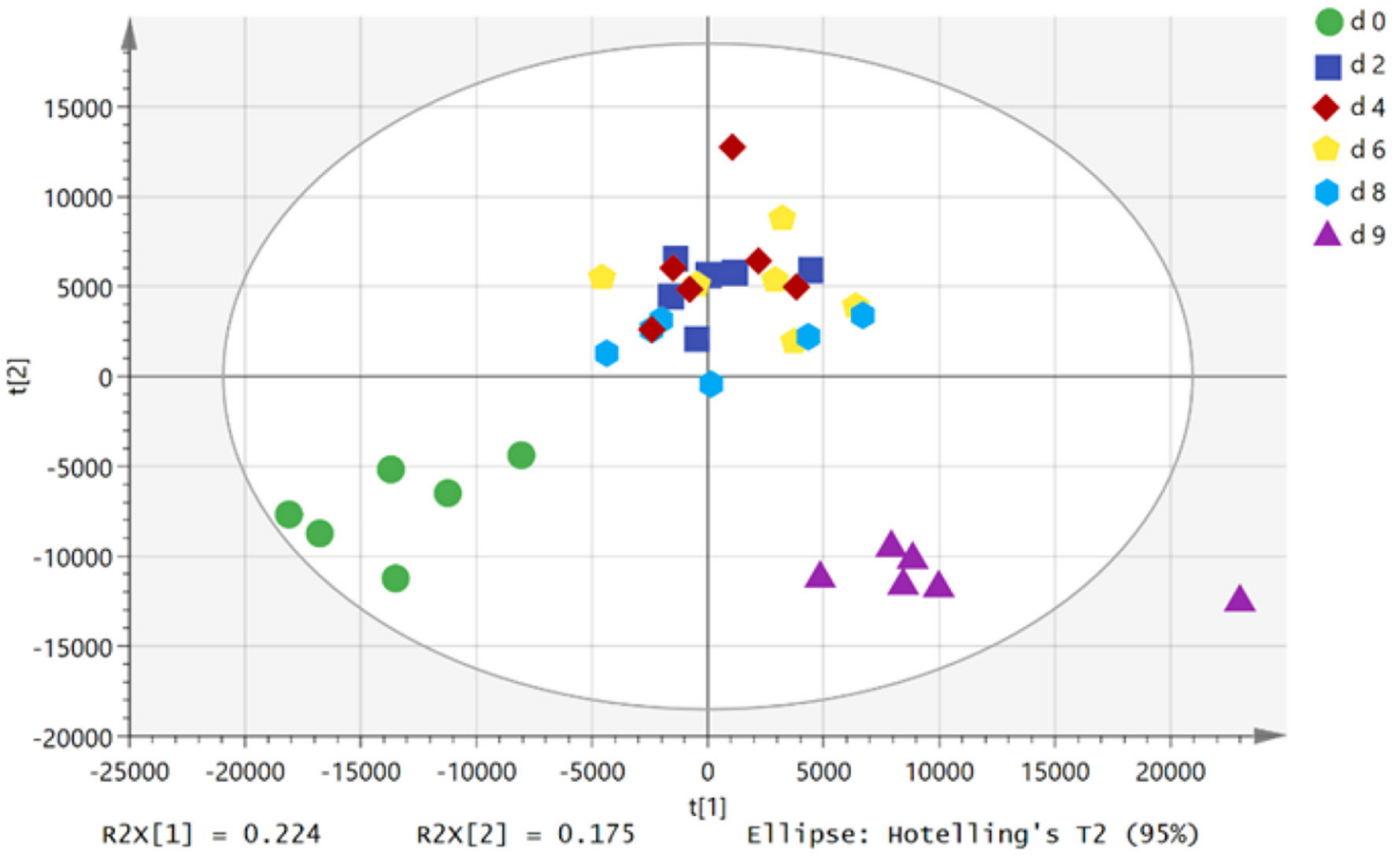
PCA score plots of plasma samples in different treatments. d 0, d 2, d 4, d 6, and d 8 represented 0th, 2th, 4th, 6th and 8th day of feeding level at metabolizable energy for maintenance; d 9, refeeding at 2,400 kJ ME/kg BW^0.6^·d^−1^ on d 9.

In the score chart of the principal component analysis (Figure 1), the plasma samples of growing pigs at the 0th day of FLM and the refeeding state showed clear clustering. PC 1 and PC 2 explained 40% of the total variances within the data. However, the plasma samples of growing pigs on different days of FLM overlap with each other, failing to show good clustering, indicating that the metabolism of growing pigs during FLM was relatively stable. With the extension of the feeding time of the diet at FLM, the plasma samples of growing pigs gradually moved from the third quadrant to the first quadrant on the score plot, indicating that the restriction of energy intake changes the body's plasma metabolites.

During the refeeding state of growing pigs, the plasma samples gradually moved from the first quadrant to the fourth quadrant, which also showed that the resumption of dietary at *ad libitum* level caused changed in the body's metabolism. Furthermore, during refeeding stage, the metabolites in the plasma samples of growing pigs returned to close the relative position at the 0th day of FLM. However, a part of metabolites in the plasma during refeeding state showed significantly difference when compared with those at the 0th day of FLM.

### The Identification of Differential Metabolites in Growing Pig Plasma During FLM

**Figure 2:**
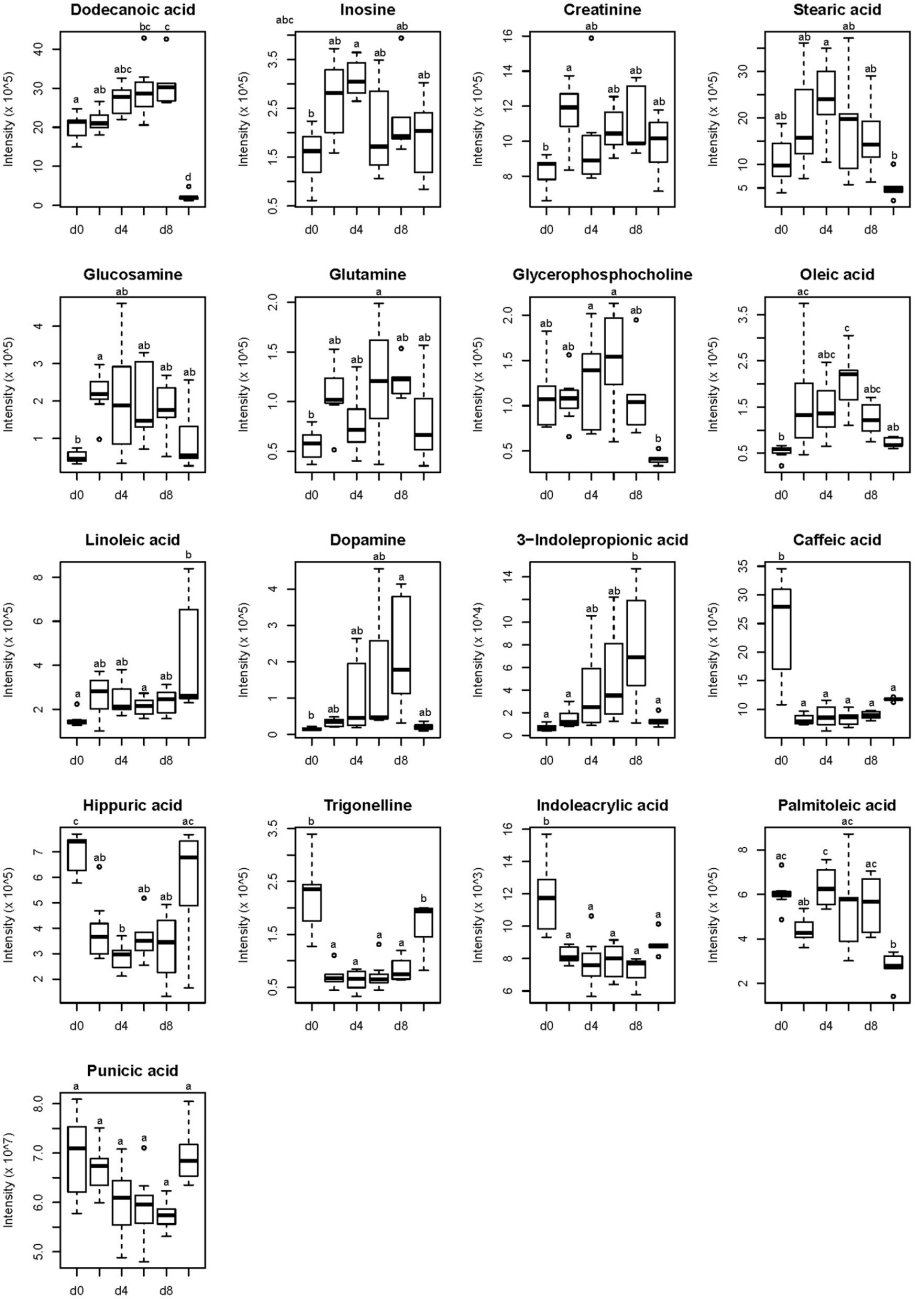
Identified compounds of growing pigs changed during feeding level at metabolizable energy for maintenance. Relative concentrations of identified compounds are presented on the y-axis. Time points of sampling are presented on the x-axis and are defined as follows: d 0, d 2, d 4, d 6, and d 8 represented 0th, 2th, 4th, 6th and 8th day of feeding level at metabolizable energy for maintenance; d 9, refeeding at 2,400 kJ ME/kg BW^0.6^·d^−1^ on d 9. ^a, b, c^ represents significant difference, *p* < 0.05.

Based on the accurate mass measurement of molecular ions and fragment ions with high resolution, 17 compounds were identified (Table 3). These metabolites belong to different metabolic classed: fatty acids, purines, amino acids and phosphocholines. Fold change was calculated by dividing the mean of normalized intensity of each plasma metabolite in the former by the mean of normalized intensity of each plasma in the latter. A fold change >1 indicates that the metabolite was up-regulated, whereas a fold change <1 indicates the metabolite was down-regulated. To illustrate the direction of changes at different time points, data were visualized in the form of boxplots. It can be seen from Figure 2 that, compared to the 0th day of FLM, the changes in the plasma of the growing pigs during FLM were mainly divided into two categories. Firstly, the relative abundances of metabolites were all up-regulated, mainly including linoleic acid, glutamine, oleic acid, creatinine and dopamine. Secondly, the relative abundance of another type of differential metabolites were all down-regulated during FLM, mainly including hippuric acid and trigonelline. At the same time, no matter how changed the plasma differential metabolites of growing pigs were under the conditions of FLM, their relative abundances were not significantly different after 4 days of feeding at FLM.

**Table 3:** Effects of feeding level at metabolizable energy for maintenance on the concentration of plasma biochemical parameters in growing pigs.

**No**.	**Metabolites**	**m/z**	**Formula**	**Fold change** ^**a**^					**Related pathway**
				**d 2/d 0**	**d 4/d 0**	**d 6/d 0**	**d 8/d 0**	**d 9/d 0**	
1	Dodecanoic acid	218.2105	C_12_H_24_O_2_	1.08	1.34	1.47	1.57	0.16	Fatty acid metabolism
2	Inosine	291.0686	C_10_H_12_N_4_O_5_	1.75	2.04	1.37	1.53	1.41	Purine metabolism
3	Creatinine	136.0476	C_4_H_7_N_3_O	1.40	1.20	1.30	1.35	1.37	Arginine and proline metabolism
4	Stearic acid	302.3040	C_18_H_36_O_2_	1.77	2.23	1.60	1.47	0.44	Fatty acid metabolism
5	Glucosamine	162.0755	C_6_H_13_NO_5_	6.01	3.96	3.92	3.42	2.78	Amino sugar and nucleotide sugar metabolism
6	Glutamine	129.0656	C_5_H_10_N_2_O_3_	1.90	1.39	2.14	2.82	1.66	Arginine and proline metabolism
7	Glycerophosphocholine	258.1090	C_8_H_20_NO_6_P	0.99	1.13	1.39	1.02	0.37	Ether lipid metabolism
8	Oleic acid	283.2618	C_18_H_34_O_2_	3.02	2.77	3.83	2.33	1.49	Fatty acid metabolism
9	Linoleic acid	281.2462	C_18_H_32_O_2_	1.71	1.64	1.39	1.54	2.78	Linoleic acid metabolism
10	Dopamine	171.1122	C_8_H_11_NO_2_	2.17	7.19	10.69	14.59	2.20	Tyrosine metabolism
11	3-Indolepropionic acid	207.1119	C_11_H_11_NO_2_	2.21	5.86	7.70	11.38	5.69	Tryptophan metabolism
12	Caffeic acid	163.0383	C_9_H_8_O_4_	0.34	0.37	0.35	0.37	0.49	Fatty acid metabolism
13	Hippuric acid	180.0649	C_9_H_9_NO_3_	0.56	0.41	0.52	0.47	0.74	Phenylalanine metabolism
14	Trigonelline	138.0544	C_7_H_7_NO_2_	0.32	0.29	0.33	0.38	0.63	Nicotinic acid metabolism
15	Indoleacrylic acid	205.0963	C_11_H_9_NO_2_	0.63	0.59	0.60	0.55	0.67	Tryptophan metabolism
16	Palmitoleic acid	272.2572	C_16_H_30_O_2_	0.47	0.67	0.56	0.59	0.28	Fatty acid metabolism
17	Punicic acid	279.2306	C_18_H_30_O_2_	0.96	0.87	0.85	1.16	1.01	Fatty acid metabolism

*^a^Fold change was calculated by dividing the mean of normalized intensity of each plasma metabolite in the former by the mean of normalized intensity of each plasma in the latter. Fold change > 1 indicates that the metabolite was up-regulated, whereas fold change <1 indicates the metabolite was down-regulated. d 0, d 2, d 4, d 6, and d 8 represented 0th, 2th, 4th, 6th and 8th day of feeding level at metabolizable energy for maintenance; d 9, refeeding at 2,400 kJ ME/kg BW^0.6^·d^−1^ on d 9*.

## Discussion

Under the condition of swine production, due to the impact of internal management or external market factors, growing and finishing pigs fed restrictively were occasionally occur. On the one hand the finishing pigs were forced to limit their feed for economic benefits or to prevent excessive fat deposition during the fattening stage; On the other hand, the animals voluntarily reduced their feed intake due to factors such as heat stress or disease (8). Regardless of the reasons for restricted feeding, pigs would adjust the corresponding energy metabolism process to maintain the body's homeostasis.

This metabolic adjustment was mainly to reduce the quality of parenchymatous organs with high turnover rates, thereby reducing energy expenditure (19). This experiment was conducted to feed growing pigs close to the state of maintain energy metabolism by feeding restriction and explored the effect of FLM on the metabolism of growing pigs.

### The Effect of FLM on Lipid Metabolism in Growing Pigs

Energy balance plays an important role in the physiological functions of mammals. Energy imbalance may lead to obesity, anorexia and other physiological or pathological reactions (20). Studies have shown that starvation or feeding restriction can lead to metabolic disorders in the animal's body, causing energy supply of some tissues to be converted from oxidized glucose to oxidized fatty acids or proteins. Moreover, regulatory factors related to the circulatory system of the body might be changed, such as reducing insulin content and increasing free fatty acid concentration (20). In the present experiment, the plasma triglyceride content was not significantly affected when compared with the 0th day of FLM, indicating that the energy supply by lipid metabolism was not significantly affected. Similarly, the concentration of total cholesterol, low-density lipoprotein, and high-density lipoprotein in plasma have no significant difference during the stage of FLM, which also indicated that the body's energy supply through lipid metabolism was relatively stable. Franczak et al. (21) studied the effect of restricted feeding on the blood biochemical parameters of pregnant sows during the embryo implantation stage. They also found that there was no significant difference between low-density lipoprotein and high-density lipoprotein in the control group and the restricted feeding group. However, the concentration of total cholesterol, low-density lipoprotein, and high-density lipoprotein decreased significantly when growing pigs fed close to *ad libitum*. This indicated that refeeding close to *ad libitum* in growing pigs caused changes in body lipid metabolism (22). Similarly, Milinkovic-Tur et al. (23) studied the effects of hunger strike and refeeding on the content and activity of antioxidant systems in chickens of different genders, and found that refeeding resulted in a decrease in plasma cholesterol. However, Hermier et al. (24) studied the changes in plasma lipoprotein profiles of high-fat breeds and low-fat breeds of chickens under hunger strike and refeeding. The results showed that low-density lipoproteins and high-density lipoproteins did not significantly affected after refeeding. Therefore, the regulatory effect of refeeding on body lipid metabolism needs further research.

The lipids in animals can be divided into triglycerides, phospholipids and steroids, and triglycerides are the most important lipids (25, 26). When the energy supply is insufficient, the animals will break down lipids to supply energy to meet energy requirement of the body. This is a strategy for the body to conserve glucose and protein, because these are the main energy-supply substances for the vital organs of the body (27).

In the present experiment, the fatty acids such as linoleic acid, oleic acid and stearic acid in the plasma of growing pigs were all up-regulated during FLM. This indicated that when growing pigs were under energy-restricted feeding, fatty acids were released into the blood by the breakdown of triglycerides stored in fat cells, thereby increasing the fatty acid content in the plasma (28). Wood et al. (29) pointed out that more than half of the fatty acids in animal lipid were unsaturated fatty acids, and the oxidation rate of unsaturated fatty acids were faster than that of saturated fatty acids. Therefore, compared with saturated fatty acids, unsaturated fatty acids were more effective energy-supply substrates, which may explain the identification of a variety of unsaturated fatty acids in the current experiment.

Trigonelline is an alkaloid that exists in many natural plants such as corn, barley, soybeans and peas (30). Studies have shown that trigonelline in the body participates in key enzymes that regulate glucose and lipid metabolism, such as glucokinase, glucose-6-phosphatase, fatty acid synthase, and carnitine palmitoyl transferase (31). In the current experiment, compared with the 0th day of FLM, the relative concentration of trigonelline in plasma was significantly reduced with the extension of FLM. After refeeding close to *ad libitum*, the relative concentration of trigonelline then returned to the level before feeding restriction. The results indicated that the synthesis of trigonelline in growing pigs was affected by the level of feed intake (32). In addition, trigonelline generates niacin through demethylation to participate in the regulation of glucose and lipid metabolism. Trigonelline and nicotinamide have also been shown to have vitamin B3 activity (33).

Therefore, the decrease in plasma trigonelline content of growing pigs during FLM may be explained that trigonelline generates nicotinic acid through demethylation and participates in the regulation of energy metabolism.

### The Effect of FLM on Amino Acid Metabolism in Growing Pigs

The response of an animal's body to feeding levels is not only manifested in changes of lipid metabolism, but also in changes of the body's amino acid metabolism (20, 22). In the current experiment, growing pigs at FLM had no significant effect on the plasma total protein and albumin concentration, while the concentration of plasma urea nitrogen was significantly reduced during the period of FLM. Wiecek et al. (22) studied the effects of feed intake and linseed oil addition on body metabolism, hormone parameters, and fatty acid composition in muscle and adipose tissue of growing pigs. The results showed that feeding restriction led to a decrease in urea nitrogen concentration, while total protein and albumin concentration in the plasma were not affected by the level of feed intake. Plasma urea nitrogen content is often used as an indirect indicator to evaluate the utilization of amino acids in diets. The decrease in blood urea nitrogen concentration indicated that growing pigs have increased the utilization of dietary nitrogen and the body's urea synthesis has decreased (34, 35). Meanwhile, Lovatto et al. (8) believed that the protein turnover of growing pigs under restricted feeding conditions was reduced, thereby improving energy utilization efficiency. This hypothesis was confirmed by finding the reduction of urine nitrogen excretion in growing pigs under restricted feeding conditions. In the present experiment, the plasma urea nitrogen level of growing pigs increased after refeeding and returned to the level before feeding restriction. Lovatto et al. (8) studied the effect of restricting feed intake and then resuming normal food intake on energy metabolism in growing pigs, and found that the urine nitrogen excretion of growing pigs was also significantly increased after refeeding. In the present experiment, the plasma albumin concentration of growing pigs was significantly reduced after refeeding. Studies have pointed out that when the protein content in tissue cells of the body decreases, circulating proteins (mainly albumin) in the blood circulatory system could be used as a source of rapid compensation of tissue cell proteins (23). Therefore, the supply of exogenous protein after refeeding was sufficient to meet the protein requirements of the body's tissues and cells, which may be the reason for the reduction of albumin concentration in the blood.

Glutamine is a rich amino acid in the blood, which plays an important role in the physiology and metabolism of tissue cells (36). One of its functions is to act as a non-toxic nitrogen carrier, providing the animal with the necessary nitrogen source and promoting the synthesis of muscle protein (37). What is more, glutamine is also one of the important substrates of the body's respiration. Glutamine can be used as a precursor for the body to synthesize glucose under the condition of insufficient external energy intake, providing a carbon source in the process of gluconeogenesis (38). The results of previous study showed that glutamine concentration of growing pigs was significantly down-regulated in the fasting state. In the current experiment, the relative plasma glutamine concentration of growing pigs during FLM was significantly increased. Manso et al. (39) explored the effects of dietary supplementation of glutamine and glutamate on the concentration of glutamine in sow milk. They also found that compared with the control group, the addition of glutamine can significantly increase the concentration of glutamine in milk and plasma of sows, while effectively prevent the decrease of muscle glutamine content. Therefore, the reason for the increase in plasma glutamine concentration of growing pigs may be that a certain amount of exogenous glutamine was provided in the diets during FLM, which increase the concentration of plasma glutamine after digestion and absorption through the gastrointestinal tract, and participates in the body's energy metabolism and nitrogen metabolism.

Dopamine is mainly formed by the hydration of its precursor amino acid tyrosine through tyrosine hydroxylation (40). Eisenhofer et al. (41) pointed out that the level of dopamine in plasma increased by more than 50 times after feeding, indicating that dopamine is mainly produced by the digestion and absorption process of animals. Many *in vitro* and *in vivo* studies have shown that dopamine in peripheral blood played an important role in energy metabolism and neurotransmitter metabolism. First, the potential functions of dopamine in digestive tract included protecting the intestinal mucosa and reducing gastrointestinal motility (42). There was evidences that dopamine can inhibit glucose-induced insulin secretion (43). Secondly, dopamine is a member of the catecholamine family and is the precursor for the synthesis of epinephrine and norepinephrine, which means that dopamine can play an important role in the immune system, kidneys, pancreas and other tissues and organs (44).

Creatinine is an irreversible non-enzymatic metabolite of creatine in animals, which is mainly excreted through the kidneys (45). Creatine is the precursor of creatine phosphate. As the energy storage substance of the body, creatine phosphate can quickly convert the stored energy into ATP to provide energy for the body when the external energy supply is insufficient or during strenuous exercise (46). Creatinine in animal blood is mainly from exogenous and endogenous sources. Exogenous creatinine refers to the ingestion and absorption of meat by animals, while endogenous creatinine is mainly the product of metabolism of muscle tissue in the body (38). Thus, to a certain extent, the concentration of creatinine in the blood of growing pigs can reflect the metabolism of nitrogen. Studies have shown that the serum creatinine concentration of Mongolian lambs was significantly higher than that of the control group after the maintenance level of nutrition is restricted, and there is no significant difference between the serum creatinine concentration and the control group after high-concentration nutrition compensation (47). In the present experiment, the plasma creatinine concentration of growing pigs was significantly increased during FLM. The possible reason was that growing pigs need to continuously break down creatine and creatine phosphate in muscles to meet energy requirement for maintenance. These metabolisms produced a large amount of creatinine into the blood (48).

Hippuric acid in mammals is mostly produced by the metabolism of glycine and benzoic acid (49). This metabolic process mainly occurs in the liver, and its biosynthesis process requires the participation of ATP and CoA (50). Therefore, hippuric acid in the body (mainly urine) is often regarded as a metabolic marker of the combination of glycine and aromatic phenolic acids (benzoic acid or salicylic acid) in the liver (50). In the current experiment, the plasma hippuric acid concentration of growing pigs decreased during FLM, and the concentration increased after refeeding, indicating that hippuric acid production in the growing pigs was affected by feed intake. Pero et al. (51) pointed out that degradation of dietary protein was one of the major sources of hippuric acid formation. In the present experiment, feeding at the level of metabolizable energy requirement for maintenance was a restricted feeding process for growing pigs. The total amount of dietary protein ingested by growing pigs was reduced, which caused a decrease in plasma hippuric acid concentration. After refeeding, the metabolic level of plasma hippuric acid in growing pigs was restored. In addition to the feed intake, hippuric acid was also produced by endogenous metabolism and intestinal microbial production, as well as the joint action of the host and intestinal microbes (52). Authors should discuss the results and how they can be interpreted from the perspective of previous studies and of the working hypotheses. The findings and their implications should be discussed in the broadest context possible. Future research directions may also be highlighted.

## Conclusions

In conclusion, the plasma urea nitrogen concentration of growing pigs was significantly reduced during FLM. Metabolomics analysis showed that the relative abundance of the identified differential metabolites such as linoleic acid, glutamine, oleic acid, creatinine, dopamine and stearic acid were up-regulated during FLM. while the relative abundances of uric acid and trigonelline were all down-regulated. Furthermore, there was no significant difference in the plasma biochemical indexes and the content of differential metabolites in growing pigs on the 4th to 8th day of FLM. Thus, the results of the study showed that the energy metabolism of growing pigs was relatively stable after 4 days of feeding at FLM.

## Data Availability Statement

The original contributions presented in the study are included in the article/supplementary material, further inquiries can be directed to the corresponding author.

## Ethics Statement

The animal study was reviewed and approved by Institutional Animal Care and Use Committee of China Agricultural University.

## Author Contributions

HL, YC, WW, and ZJ contributed to conception, design of the study, and performed the experiments. HL provided the statistical analysis. XM and FW conducted writing—reviewing. HL and FW acquired the funding. All authors contributed to the article and approved the submitted version.

## Funding

This research was financially supported by the National Natural Science Foundation of China, grant numbers 32102587 and 31372317, the National Key Research and Development Program of China, grant number 2021YFD1300201, Natural Science Foundation of Shandong Province of China, grant number ZR2021QC016, and the 111Project, grant number B16044.

## Conflict of Interest

The authors declare that the research was conducted in the absence of any commercial or financial relationships that could be construed as a potential conflict of interest.

## Publisher's Note

All claims expressed in this article are solely those of the authors and do not necessarily represent those of their affiliated organizations, or those of the publisher, the editors and the reviewers. Any product that may be evaluated in this article, or claim that may be made by its manufacturer, is not guaranteed or endorsed by the publisher.
